# Inkjet Printing Infiltration of the Doped Ceria Interlayer in Commercial Anode-Supported SOFCs

**DOI:** 10.3390/nano11113095

**Published:** 2021-11-16

**Authors:** Rumen I. Tomov, Thomas B. Mitchel-Williams, Eleonora Venezia, Michal Kawalec, Mariusz Krauz, Ramachandran Vasant Kumar, Bartek A. Glowacki

**Affiliations:** 1 Department of Materials Science & Metallurgy, University of Cambridge, Cambridge CB3 0FS, UK; tbmwilliams@gmail.com (T.B.M.-W.); eleonora.venezia92@gmail.com (E.V.); rvk10@cam.ac.uk (R.V.K.); bag10@cam.ac.uk (B.A.G.); 2 Institute of Power Engineering—Research Institute, Mory 8, 01-330 Warsaw, Poland; kawalec@cerel.pl (M.K.); krauz@cerel.pl (M.K.)

**Keywords:** solid oxide fuel cells, inkjet printing, infiltration, doped ceria, cobalt oxide

## Abstract

Single-step inkjet printing infiltration with doped ceria Ce_0.9_Ye_0.1_O_1.95_ (YDC) and cobalt oxide (Co_x_O_y_) precursor inks was performed in order to modify the properties of the doped ceria interlayer in commercial (50 × 50 × 0.5 mm^3^ size) anode-supported SOFCs. The penetration of the inks throughout the La_0.8_Sr_0.2_Co_0.5_Fe_0.5_O_3−δ_ porous cathode to the Gd_0.1_Ce_0.9_O_2_ (GDC) interlayer was achieved by optimisation of the inks’ rheology jetting parameters. The low-temperature calcination (750 °C) resulted in densification of the Gd-doped ceria porous interlayer as well as decoration of the cathode scaffold with nanoparticles (~20–50 nm in size). The I–V testing in pure hydrogen showed a maximum power density gain of ~20% at 700 °C and ~97% at 800 °C for the infiltrated cells. The latter effect was largely assigned to the improvement in the interfacial Ohmic resistance due to the densification of the interlayer. The EIS study of the polarisation losses of the reference and infiltrated cells revealed a reduction in the activation polarisations losses at 700 °C due to the nano-decoration of the La_0.8_Sr_0.2_Co_0.5_Fe_0.5_O_3−δ_ scaffold surface. Such was not the case at 800 °C, where the drop in Ohmic losses was dominant. This work demonstrated that single-step inkjet printing infiltration, a non-disruptive, low-cost technique, can produce significant and scalable performance enhancements in commercial anode-supported SOFCs.

## 1. Introduction

Commercial solid oxide fuel cells (SOFCs) provide highly efficient energy conversion, simultaneously offering the additional benefit of combined heat and power generation. One of the attractive properties of high-temperature SOFCs is the ability to use hydrocarbon fuels directly through internal reforming within the fuel cell stack. Such SOFCs typically operate at high temperatures (800–1000 °C) allowing for fuel flexibility [1,2,3,4,5,6,7,8]. One of the important technical challenges operating in this temperature range is the undesirable deterioration and long-term instability due to the chemical interaction between the dissimilar components of the fuel cell stack [9,10]. A common approach toward resolving the latter issue is replacing the classical La_1-x_Sr_x_MnO_3−δ_ (LSM) cathode with mixed ion–electron conductors (MIECs), which were reported to have higher tolerance toward Cr species compared to LSM electrodes [11,12]. Beneficially, the introduction of MIECs as cathode materials results in an enlargement of the surface active areas involved in the oxygen reduction reaction (ORR [13]). Various MIEC materials, such as La_1-*x*_Sr*_x_*CoO_3−δ_ (LSC) [14], La_1-*x*_Sr*_x_*FeO_3−δ_ (LSF) [15] or La_1-*x*_Sr*_x_*Co_1-*y*_Fe*_y_*O_3−δ_ (LSCF) [16], have been experimented with extensively. LSCF is often used as a material of choice due to a combination of high electronic conductivity and high oxygen ionic conductivity. However, MIECs were found to react with Y-stabilised zirconia (YSZ) electrolytes during cell processing, forming deleterious secondary phases, such as SrZrO_3_ and La_2_Zr_2_O_7_ [17]. Moreover, substantial differences in thermal expansion coefficients (TECs) between the MIECs and commercial electrolytes could lead to thermal cycling instability and short SOFC lifetimes [18]. An introduction of a diffusion barrier layer (interlayer) of doped ceria between the YSZ electrolyte and the MIEC cathode is commonly used to alleviate the issue [19,20]. Such interlayer has to be thin and dense in order to avoid the addition of an additional Ohmic resistance. Vacuum techniques, such as pulsed laser deposition [21] and magnetron sputtering [22], have been successfully used to deposit efficient dense doped ceria barrier coatings. Unfortunately, the use of vacuum techniques in SOFC processing is considered prohibitively expensive. Commercial SOFCs most often use doped ceria interlayers prepared by the conventional screen-printing technique. The sintering temperatures of the as-deposited interlayer have to be kept below the electrolyte sintering temperatures in order to prevent the formation of an undesirable (Zr_1-x_Ce_x_)O_2−y_ solid solution [23]. This temperature limitation, along with the difference in TECs of doped ceria and doped zirconia, often lead to the formation of porous interlayers. Consequently, diffusion of Sr species is commonly observed, reaching and reacting with the YSZ electrolyte [24]. To resolve the problem, significant efforts were spent on wet nano-chemistry methods, delivering an improved interlayer deposition, e.g., spin coating [25], dip coating [26], spray pyrolysis [27] and electrostatic spray pyrolysis [28], all producing doped ceria interlayers with various degrees of densification. Inkjet printing of suspension inks was also successfully applied for the fabrication of cathode interlayers (LSCF–GDC) [29] as well as a NiO-YSZ_ anode interlayer [30]. Although offering scalability, the above-mentioned techniques often need multistep processing with repeated heat treatments at relatively low deposition rates. The wet-chemical methods, with the exception of inkjet printing, also lead to a certain degree of non-uniformity and are inefficient in terms of ink losses outside the cell area.

Another problem observed at high operational temperatures is the compositional degradation of MIEC cathodes [31,32,33,34]. The effect is commonly ascribed to Sr enrichment of the surface, where insulating SrO, (Sr(OH)_2_ and SrCO_3_ species form and subsequently suppress oxygen surface exchange kinetics [35]. As demonstrated by Rupp et al. [36], a sub-monolayer coverage (~4% of a SrO monolayer) on the LSC thin-film electrode leads to severe deactivation, while a minor decoration with Co oxide (~2% of a monolayer) enhances the oxygen exchange rate by ~13%. The co-existence of A- and B-site metal atoms on the surface was shown to have a strong influence on the ORR, with B-site metal atoms (Co or Fe) being more reactive than A-site atoms (La or Sr) [37]. Nano-engineering of perovskite cathodes by infiltration has been actively researched in recent years. Improved performances and stability of the LSCF-based cathodes were demonstrated via decoration of the porous cathode scaffolds with nanoparticles (e.g., noble and transition metal oxides, doped and un-doped ceria, MIEC compounds, etc.). Nano-decoration of screen-printed La_0.8_Sr_0.2_Co_0.5_Fe_0.5_O_3−δ_ (LSC8255) with Gd_0.2_Ce_0.8_O_2_ [38] and tape-casted La_0.6_Sr_0.4_Co_0.2_Fe_0.8_O_3−δ_ (LSCF6428) cathodes with Sm_0.2_Ce_0.8_O_1.95_ (SDC) [39] was reported to reduce the polarisation resistance between two and four times at 750 °C. Dual infiltration of both doped ceria and Co_x_O_y_ inks was shown to deliver a synergetic effect, improving both the catalytic activity as well as the performance durability via suppression of the Co_x_O_y_ nanoparticle aggregation [40]. Co_x_O_y_ nanoparticles were found to serve multiple functions—to act as a sintering aid, improving the densification of doped ceria [41,42], to accelerate adsorption–dissociation–surface exchange reactions of oxygen and to suppress Sr precipitation on the surface of LSCF cathodes [43]. 

This study focuses on the use of dual infiltration via a non-disruptive scalable technique, namely inkjet printing infiltration (IJI), for improving the performance of commercial anode-supported SOFCs (50 × 50 × 0.5 mm^3^ in size). Yttrium-doped ceria (YDC) ink was jetted in nano-litre-volume drops impinging with high velocity on the surface of the LSCF cathodes. The momentum of the drops forced a sufficient amount of ink to reach the interface between the electrolyte and the cathode, depositing it into the pores of the interlayer. A second infiltration with Co_x_O_y_ ink was applied in order to promote the sintering behaviour of the infiltrated doped ceria as well as to enhance the catalytic activity of the infiltrated cathode scaffold. The influence of such nano-infiltration on the electrochemical performance was studied, taking into account the changes in both the Ohmic resistance of the Ce_0.9_Gd_0.1_O_1.95_ (GDC) interlayer and the polarisation resistance of the LSCF cathode. A single-step infiltration, leading to low overall infiltrate-loading levels, was implemented in order to avoid blocking of the porous gas channels and introduction of additional concentration polarisation losses.

## 2. Materials and Methods

The choice of inks and infiltration technique used in the experiment was based on our previous works on the synergy effect of dual infiltrations in MIEC cathodes [40,43,44]. Such dual infiltration (e.g., doped ceria and Co_x_O_y_) was shown to enhance the MIEC cathode performance and improve the long-term stability via suppression of Sr segregation. The goal of this work was to achieve a non-disruptive transfer of the IJI to commercial SOFC processing, contributing to the densification of the doped ceria interlayer and the enhancement of the cathode performance. 

Adversely, overloading of the porous cathode scaffold with infiltrate ink is prone to lead to an increase in concentration losses as well as to effective degradation of the LSCF cathode performance. The latter is caused by the partial masking of the active surface area by nano-decorations possessing lower catalytic efficiency. Thus, we attempted to achieve the desired SOFC performance enhancement using minimum amounts of inks delivered by the IJI method with high precision and uniformity as well as no wastage.

**Figure 1:**
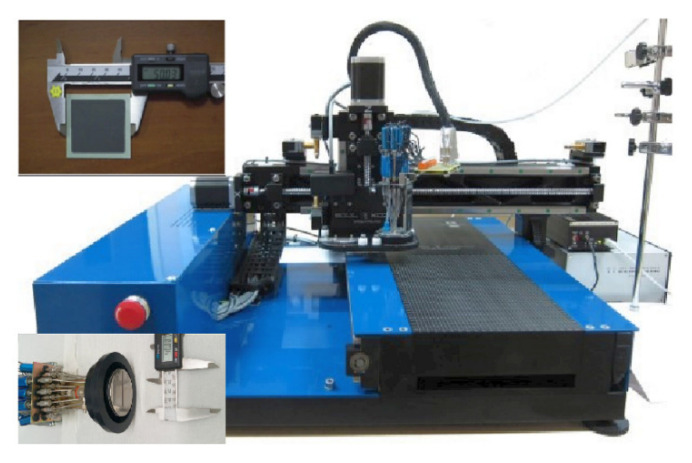
Customised inkjet printing system used for the infiltration experiment (the insets show the 16-nozzle Domino valve-jet print head and the commercial anode-supported SOFC).

The infiltration of active precursors in SOFC electrodes is commonly done in multiple steps (depending on the desired loading levels) followed by low-temperature (<800 °C) calcination. To achieve the desired loading, deeper penetration and more uniform distribution, some research groups perform vacuum treatments after each infiltration step. The infiltrate inks are also often tailored with surfactants and gelling agents in order to attain control over the phase and morphology of the nano-decoration—particle size, distribution, coverage, etc. Tuning the wetting properties of the ink with organic solvents is another efficient way to achieve uniform nano-decoration after calcination. The infiltration procedures reported in the literature have been predominantly performed in a laboratory environment using sample immersion or micro-pipetting. Such procedures are wasteful, slow and not scalable. Several attempts to scale up the process, making it feasible for conventional SOFC technology, have been reported. Lee et al. [45] implemented foam rollers to introduce the inks in the porous anode functional layer. Kiebach et al. [46] “flashed” the infiltrate metal nitrates solutions through the manifold compartments of the SOFC stacks. Spraying the cathode surface with an atomising nozzle was successfully reported by Dowd et al. [47]. All of these approaches achieved scalability at the expense of significant amounts of wasted ink, while having limited control over the uniformity of the infiltration process. In contrast, the inkjet printing infiltration method offers uniform delivery of precisely positioned small droplets (nano-litre volumes) at high rates (kHz) over large surfaces with high lateral resolution. It is inherently cost effective and environmentally friendly due to minimisation of the ink amount used and the lack of wastage. Commercial inkjet printing systems are widely available, ranging from laboratory-scale systems to industrial high-throughput machines. A scalable, low-cost, uniform infiltration was reported by Mitchell-Williams et al. [48] using inkjet printing to infiltrate GDC into 8YSZ SOFC anodes. Attempts to use commercial valve jet technology for the production of SOFC components (anodes and electrolytes) was reported by Tomov et al. [49] and Wang et al. [50]. Tomov et al. [43] also used inkjet printing infiltration to infiltrate GDC into LSCF/GDC composite cathodes. Figure 1 illustrates the customised inkjet printing system, developed in-house, allowing for printing over a large surface area with high throughput. The 16-nozzle Domino valve-jet print head used in this work is shown in the inset. The effective width of the printed single pass was ~40 mm. The commercial anode-supported SOFC shown in the second inset had an effective cathode area of 40 mm × 40 mm, which allowed for completing the infiltration run with a single pass of the print head. 

## 3. Experimental Section

### 3.1. Anode-Supported SOFC Preparation

Commercially available anode-supported SOFCs (CEREL, Boguchwała, Poland) with screen-printed LSCF cathodes were infiltrated by IJI with doped ceria and cobalt oxide precursor inks. The anode supports were made using high-pressure injection-moulding. A functional layer (8YSZ+NiO—1:1) and an electrolyte layer (8YSZ) were applied using screen printing. After drying, the coatings were sintered at 1400 °C for 3 h. Then, a GDC interlayer was deposited by screen printing and sintered at 1350 °C for 1h. A La_0.6_Sr_0.4_Co_0.2_Fe_0.8_O_3−δ_ cathode (LSCF thickness ~25 µm, porosity ~25 vol%) was screen-printed and sintered at 1100 °C for 1 h. In the as-prepared 50 × 50 × 0.5 mm^3^ cells, a 5-μm-thick YSZ electrolyte was supported by a Ni-YSZ cermet anode (including an anode functional layer). The LSCF cathode with an effective area of 16 cm^2^ was separated from the electrolyte by a thin GDC interlayer (~2–3 μm thick). A current-conducting LSM coating was applied on the top of LSCF before testing. A detailed description of the anode-supported SOFC manufacturing can be found elsewhere [51].

### 3.2. Infiltrate Ink Synthesis

Ce_0.9_Ye_0.1_O_1.95_ (YDC) precursor solutions were prepared by diluting stoichiometric quantities of cerium nitrate hexahydrate (99.999%, Alfa Aesar, Heysham, UK) and yttrium nitrate hexahydrate (99.9% Alfa Aesar) in absolute ethanol. Urea (>99.5%, Thermo Fisher Scientific, Heysham, UK) was added as a complexing agent in a 1:1.5 molar ratio (metals:urea). For the Co_x_O_y_ ink, the precursor ink was prepared in a similar manner using cobalt nitrate hexahydrate (99%, VWR, Lutterworth, UK) and urea. The powders were dissolved with stirring and heating at 40 °C. The solutions were cooled to room temperature before being passed through a 3 μm glass fibre filter.

### 3.3. Jetting Optimisation

The jetting behaviour of a print-head nozzle is generally described with the help of dimensionless Reynolds (*Re*), Weber (*We*) and Ohnesorge (*Oh*) numbers [52]: $Re=\frac{\rho u_{0}r_{0}}{\mu }$; $We=\frac{\rho u_{0}{}^{2}r_{0}}{\gamma }$; and $\;Oh=\frac{We^{0.5}}{Re}$, where *u_o_, r_o_, ρ, μ* and *γ* denote the drop velocity, drop radius, ink specific mass density, ink viscosity and surface tension coefficient, respectively. Derby et al. [53,54] defined the optimal condition of jetting without splashing or formation of satellite drops as 20 > Z > 1 (where Z = 1/Oh). Tuning the jetting parameters to optimum values requires knowledge of the drop velocity and the drop volumes specific for any particular ink and print head. The use of an integrated drop visualisation system allows examination of the drops volumes and velocities and assessment of the correct working parameters window. Such optimisation for the electromagnetic (valve jet) print heads was done by varying the opening times and nozzle pressures using an in-house-developed visualisation system, as described elsewhere [48]. This allowed us to achieve optimal drop formation and nearly identical loading for each of the inks used. 

According to the numerical model developed by Reis et al. [55] spreading and penetration of the drop into a porous medium is governed by a set of parameters, including the Reynolds number (*Re*), Weber number (*We*), porosity (*ε*) and contact angle (*θ*). Thus, ink penetration (depth and lateral spreading) is a complex function of fixed parameters (*ε*), parameters with variability restricted by the rheological window of stable jetting for a particular ink (*ρ, μ, γ and θ*) and sufficiently variable parameters (*u_o_, r_o_*). Hence, in this study, optimisation of u_o_ and r_o_ was performed, aiming at the formation of small nano-size drops with similar volumes and impingement velocity while simultaneously preserving stable jetting without the formation of satellites or splashing. The uniformity of the drop delivery and cumulative volume of ink infiltrated was governed by the choice of the X-Y step size and the drops’ overlap distance. 

### 3.4. Inkjet Printing Infiltration

**Figure 2:**
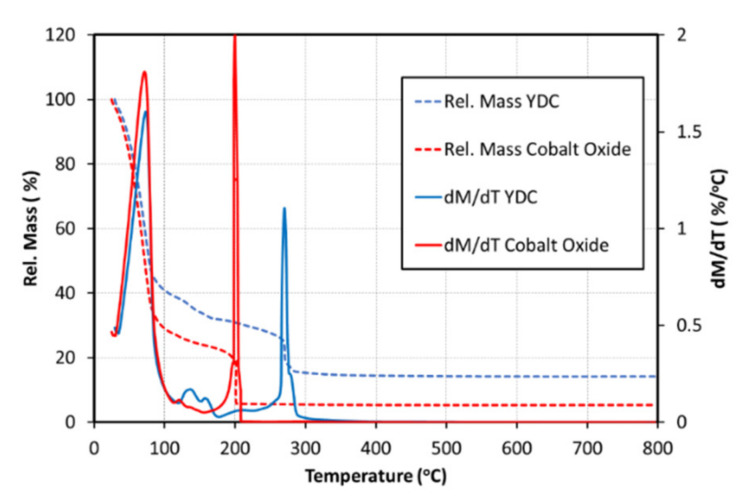
TG curves of 0.75 M YDC and Co_x_O_y_ precursor inks at a 10 °C min^−1^ heating rate in air. The final weight losses were used in thickness calculations. Temperature derivatives from the TG analysis clearly pinpoint the temperature range of mass loss.

The cathodes of the AS-SOFC (NiO-8YSZ/8YSZ/GDC/LSCF) were infiltrated by inkjet printing of the precursor inks using a commercial 16-nozzle Domino Macrojet print head. The infiltrations were performed using optimised jetting parameters for each ink, as shown in Table 1. The inks were deposited onto the cathode surface at room temperature with drop volumes of approximately ~60 nL and drop velocities of ~1.5 m s^−1^. Several infiltration passes were performed across the entire surface in a square array pattern with a spacing of 1 mm between drops. The cells were dried between the infiltration passes at temperatures high enough to remove the ink solvent but lower than the nitrate salts’ decomposition point. Thus, the permeation channels were freed for the next ink portion to be absorbed into the porous scaffolds. Figure 2 shows the TGA decomposition data for the YDC and Co_x_O_y_ precursor inks (solution, dispersant and metal nitrates).

Temperature derivatives from the TG analysis (DTG) clearly show that the biggest mass loss occurs at ~73–76 °C where ethanol solvent from both inks is liberated. Above 200 °C, further decomposition results in oxide formation and the release of gases, such as CO and CO_2_. There is no further loss observed beyond 300 °C. After the infiltration of the YDC precursor ink, the cells were heated to 500 °C for 30 min with heating and cooling rates of 5 °C min^−1^. The infiltration of the second ink (Co_3_O_4_ precursor ink (62 nL, 1.6 m s^−1^)) was performed in an identical manner. Samson et al. [56] estimated temperatures above 650 °C as required for the formation of stable Co_x_O_y_ nanoparticles. Due to the necessity to preserve a stable nanostructured interface during testing at temperatures above 700 °C, the final-stage calcination was done at 750 °C in air for 30 min (heating and cooling rates of 5 °C min^−1^). The mass loading of each infiltrate was calculated from the weight measurements before and after each infiltration cycle. Loading levels of ~5 wt% with respect to the LSCF electrode was adjusted for both YDC and cobalt oxide infiltrates. 

### 3.5. Characterisation

The electrochemical performances of the infiltrated and reference AS-SOFCs were tested in a single cell test rig (I–V and electrochemical impedance spectroscopy (EIS) - LB2000 current load and VMP3 potentiostat, BioLogic, Seyssinet-Pariset, France) at temperatures of 700 and 800 °C, with pure H_2_ flow of 1.0 NL/min acting as fuel feed and standard air flow of 2.0 NL/min as cathode feed. High-resolution SEM-EDX (Nova NanoSEM, Thermo Fisher Scientific, Waltham, MA, USA) with an acceleration voltage of 15 kV was used for microstructural analysis of the fractured cell’s cross sections. Cross sections of the cells were used for elemental mapping. All SEM-EDX analyses presented below were performed post-mortem after completion of the electrochemical testing. Surface chemistries of the bulk and the interfacial areas of the cathodes were characterised using high-resolution X-ray photoelectron spectroscopy (XPS) of wedge-polished samples (K-Alpha^+^ X-ray photoelectron spectrometer, Thermo Fisher Scientific, Waltham, MA, USA). Details about the XPS setup and measurement methodology are reported elsewhere [40].

## 4. Results and Discussion

**Figure 3:**
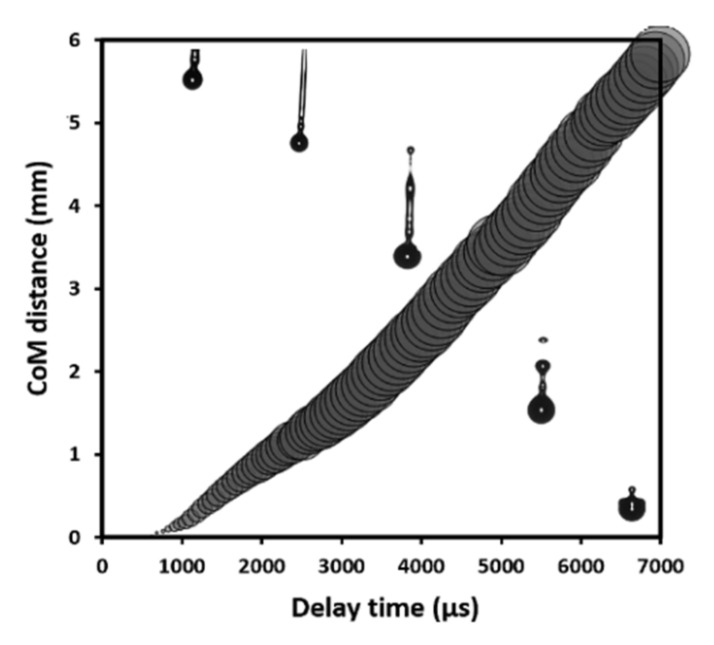
Dependence of the centre of mass (CoM) of jetted drops vs. flight delay time superimposed with the relevant visualisation drop images for the YDC ink.

Drop visualisation optimisation enabled ink-jetting parameters to be tailored in such a way that each triggering event resulted in a single drop of ink, without the formation of satellites. Figure 3 shows the dependence of the centre of mass (CoM) of YDC-EtOH ink drops on the flight delay time. Images of drops superimposed to the relevant delay times showed optimised jetting behaviour. At optimised conditions (200 mbar pressure and an opening time of 240 µs), jetting of a single drop starts with an elongated tail after detaching from the nozzle, which further merges with the main drop and forms a single drop with ~57 nL volume and ~1.5 m s^−1^ velocity. The CoM in Figure 3 was defined by the imaging software as a centre of mass of the total jetted volume, assuming an ideal symmetry of the drops (or drops) and the tail ejected. The gradually changing size of the circles in Figure 3 illustrated the increasing diameter of the main drop as a result of its merging with the tailing drops into one single volume. Further information about the jet optimisation procedure can be found in the Supplementary Materials as well as in [48]. The descripted optimisation procedure allowed to maximise the drop velocity by adjusting the drop volumes in such a manner that no splashing or satellite drop formation was observed at optimised conditions. No satellite drop formation was observed for both inks, which led to a uniform lateral distribution of the infiltrates. Z numbers of ~10.4 and ~9.7 were calculated for YDC and Co_x_O_y_ inks, respectively. Such Z numbers correspond well with the stable printability window defined by Liu et al. [54] in the region of 1 < Z < 20.

The differences in the electrochemical performances of the reference and infiltrated cells were expected to be associated with changes in (i) Ohmic (*R_s_*) resistance related to densification of the GDC interlayer, (ii) polarisation (*R_p_*) resistance associated with modification of the catalytic properties of the bulk LSCF scaffold and (iii) introduction of catalytic properties by the infiltrated Co_x_O_y_ nanoparticles. 

**Figure 4:**
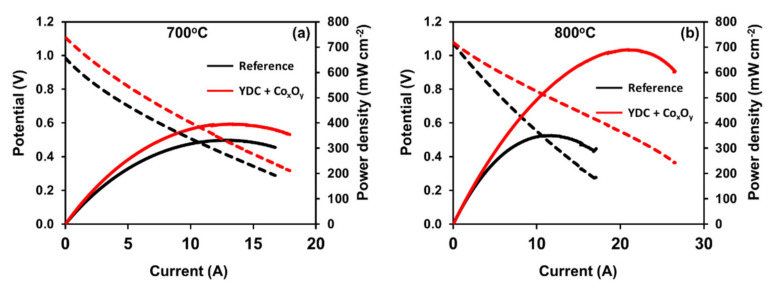
I–V curves and power densities of infiltrated and reference cells measured at (**a**) 700 °C and (**b**) 800 °C.

**Figure 5:**
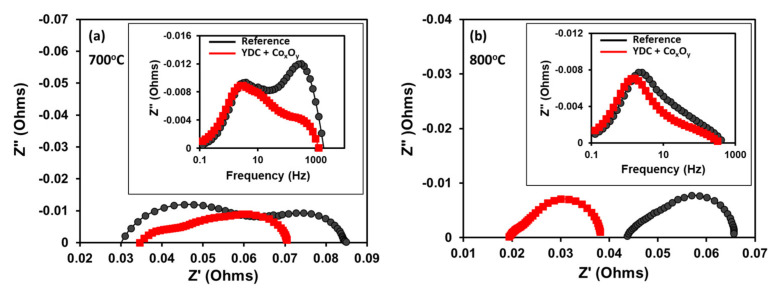
EIS Nyquist plots of infiltrated and reference cells recorded at (**a**) 700 °C and (**b**) 800 °C (the insets in the figures represent the Bode plots at relevant temperatures).

Figure 4 presents the current–potential and current–output power curves measured on the reference and infiltrated cells at 700 °C and 800 °C. Note that while at 800 °C, the open-circuit voltages (OCVs) of both cells (reference and infiltrated) cells were equal to 1.1V, at 700 °C, the OCV of the reference cell was lower (approximately 0.98 V). The infiltrated cells showed substantially improved performances in comparison to the reference cell at both temperatures. At 700 °C, the maximum output power increased by approximately 20%, from 330 to 395 mW cm^−2^. The maximum output power density at 800 °C increased from 350 mW cm^−2^ for the reference cell to 690 mW cm^−2^ for the infiltrated cells. This corresponds to a maximum power density gain of 97% as a result of the infiltration procedure. The EIS data at the OCV for the reference and infiltrated cells recorded at both temperatures is presented in Figure 5. All Nyquist plots had similar shapes consisting of overlapping suppressed semi-circular arcs. As far as the anodes of both cells were identical, the differences between the recorded spectra were ascribed to changes introduced in the cathode and the interlayer by the infiltration procedure. The Ohmic (*R_s_*) resistances were estimated from the intercept of the high-frequency arm of the Nyquist plot with the real axis, while the polarisation resistance (*R_p_*) was estimated as a difference between the low- and high-frequency intercepts. As demonstrated in Figure 5a at 700 °C, one could observe a drop in the polarisation losses (*R_p_*) related to the substantially lowered high-frequency contribution. Such improvements have been previously assigned to the enhancement of a charge transfer reaction at the extended by the infiltration LSCF/electrolyte interface [40,57]. The Ohmic resistance of the infiltrated cells was similar to that of the reference cell. The data collected at 800 °C suggested a different effect. Although a reduction in *R_p_* was observed for the infiltrated cells, it was marginal (see Figure 5b). The shapes of the Bode curves of the reference and infiltrated cells were similar (see the inset in Figure 5b) with a small reduction in Z” at lower frequencies (1–100 Hz) most likely due to enhancement of the surface exchange reaction. This equalisation could be ascribed to the enhanced ORR efficiency of the native LSCF cathode at 800 °C. Thus, the observed substantial power output improvement of infiltrated cells at 800 °C could be ascribed to the reduction in *R_s_*, as demonstrated in Figure 5b. 

Looking for proof that the infiltration of CYO and Co_x_O_y_ led to densification of the interfacial GDC interlayer, we performed post-mortem cross-sectional morphological and compositional examination of the cells’ cathode bulk as well as cathode–electrolyte interface areas by high-resolution SEM-EDX and XPS.

**Figure 6:**
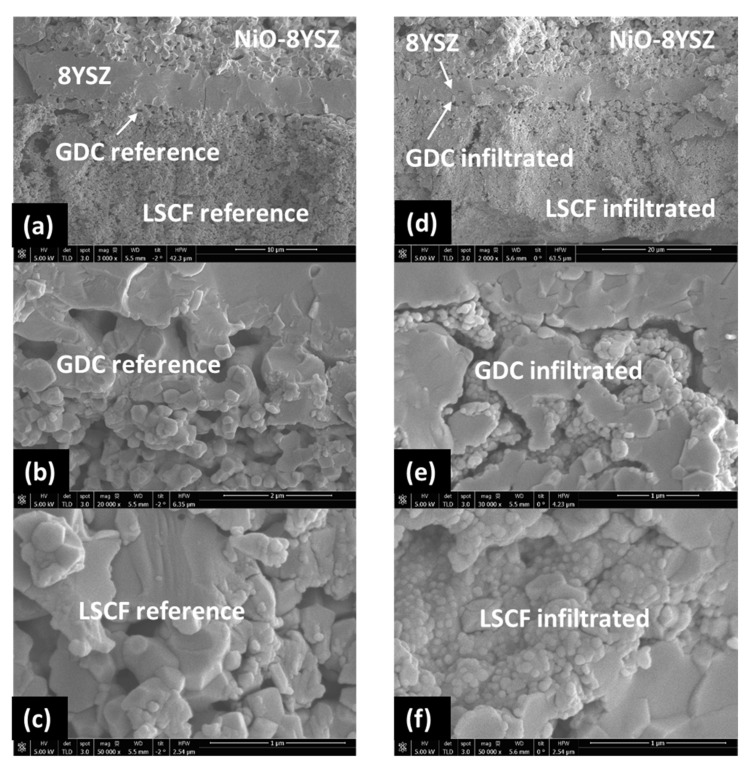
Post-mortem SEM cross-sectional images of the reference cell (**a**–**c**) and the cell infiltrated with YDC and Co_x_O_y_ (**d**–**f**). Please note that (**a**,**d**) are low-resolution images of the cross sections of the anode-supported SOFCs, (**b**,**e**) are high-resolution images of the GDC interlayer interface areas and (**c**,**f**) are high-resolution images of the LSCF bulk areas. (Note also that the bar sizes on the images signify the following—for image (**a**), 10 µm; for image (**b**), 2 µm; for image (**c**), 1 µm; for image (**d**), 20 µm; and for images (**e**,**f**), 1 µm.)

**Figure 7:**
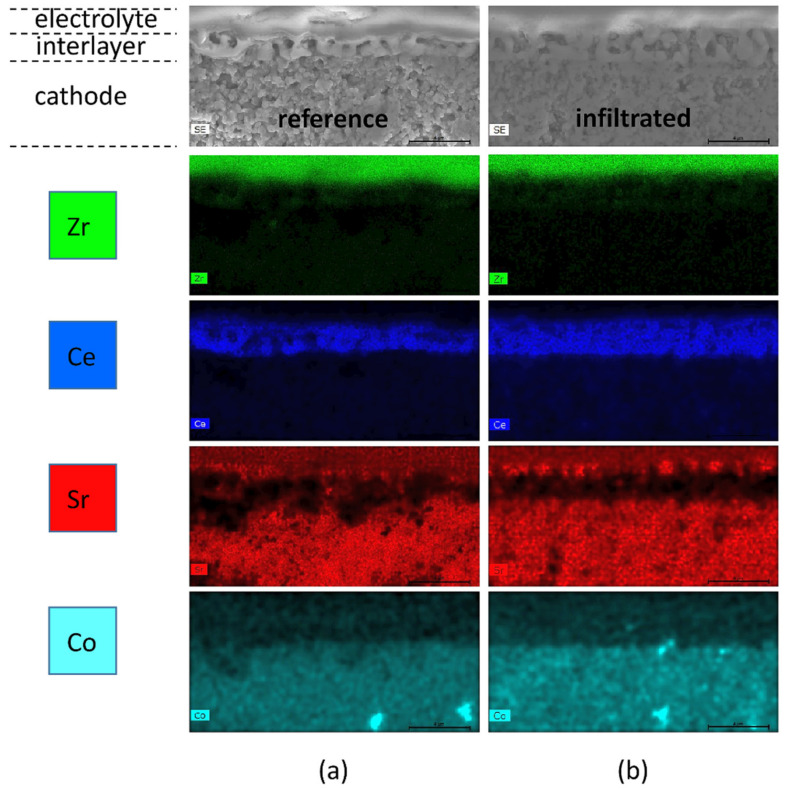
EDX mapping of the interlayer areas for (**a**) the reference cell and (**b**) the infiltrated cell. (Note that the bar size for all images is 4 µm.)

The post-mortem SEM cross-sectional images of the reference and YDC+Co_x_O_y_ infiltrated commercial cells (see Figure 6) confirmed the densification of the GDC interlayer by the infiltrated nanoparticles, which led to a significant reduction in the interlayer Ohmic resistance contribution. Figure 6a,c depicts the microstructure of the reference and infiltrated cell cross sections of the cathode/interlayer/electrolyte interfaces at lower magnification. The functional interlayer (~2–3 µm thick) was composed of GDC grains (~1.0–1.5 µm in diameter) with ~50% porosity. The concentration of infiltrated phases in the interface region was clearly visible, revealing that the interlayer was substantially densified after testing at 800 °C (see Figure 6b,e). The images from the area in the middle of the LSCF bulk (see Figure 6f) showed the infiltrated cathode bulk decorated with significantly lower decoration density, consisting of non-percolating nanoparticles. No such nano-decorations were visible on the surface of the non-infiltrated reference cell—see Figure 6c. This indicated that a substantial part of the infiltrate ink penetrates through the porous LSCF scaffold, reaching the GDC interlayer, while some of the ink decorates the surface of the cathode. The EDX maps of the interface areas in the reference and YDC-infiltrated cells are presented in Figure 7a,b, respectively. One can detect the migration of Sr to the surface of the YSZ electrolyte in both cases. A more localised presence of Sr was seen in the case of the infiltrated cells. 

**Figure 8:**
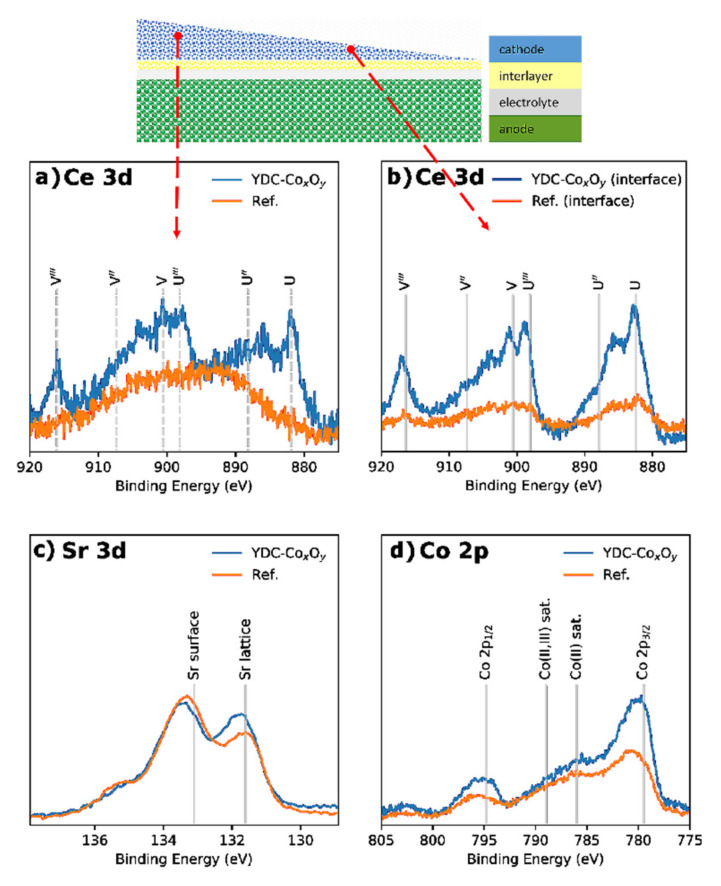
XPS spectra of Ce 3d (**a**), Sr 3d (**c**) and Co 2p (**d**) core levels of the LSCF bulk area and Ce 3d core level (**b**) of the near-interface areas for the reference and YDC-Co_x_O_y_ infiltrated cells.

XPS analysis of the bulk and near-interface LSCF areas for the commercial reference and infiltrated cells was performed by careful wedge-type polishing with diamond paste (1 µm), as shown in the inset of Figure 8. Figure 8a–d illustrates the background-corrected core-level XPS spectra of the Ce 3d (a,b), Sr 3d (c) and Co *2p* (d) bands. The intensity was normalised to the area of the La 3d_5/2_ band. The Ce 3d spectra (Figure 8a,b) showed mainly cerium-doped oxide in the +4 oxidation state, with the characteristic three doublet contributions, namely (v,u), (v”,u”) and (v”’,u”’), at positions in good agreement with the literature [58]. As expected, no doped ceria was detected in the reference LSCF bulk cathode (with observable Co, Fe and La auger peaks at the Ce 3d position), while some amount was registered in the LSCF bulk of the YDC-infiltrated LSCF (La 3d_5/2_: Ce 3d = 80:20). In contrast, at the interface of the infiltrated electrode, a much higher concentration of doped ceria was registered—La 3d_5/2_: Ce 3d = 11:89. The later observation suggested that a larger portion of the YDC ink was delivered to the area of the GDC/LSCF interface. Additionally, the measured Sr 3d core levels (see Figure 8c) suggested that the amount of Sr segregated on the cathode surface (detected as Sr(OH)_2_ and SrCO_3_ species) was reduced by the infiltration—Sr 3d_5/2_ lattice: surface = 43:57 for the reference cell and 59:41 for the infiltrated cells. A similar effect of Sr segregation suppression was observed previously by inkjet printing infiltration of GDC into composite LSCF/GDC cathodes in symmetric SOFCs [43]. A comparison of the Co 2p core-level spectra between the reference and infiltrated cells is presented in Figure 8d. Both cells exhibited a spectra of a mixed Co(II) oxide and Co(III) oxide structure, with the main Co 2p_3/2_ peak located at a BE of 779.4 eV and visible shake-up satellite structures around 786 eV and 789 eV indicative of a mixed Co(II) and Co(III) oxide. The excess of Co in the infiltrated sample was also clearly observable. 

The presented analyses correlated with the hypothesis that the majority of the infiltrated ink reaches the interface area and the resulting densification of the GDC buffer layer leads to the observed drop in the Ohmic resistance at 800 °C. This demonstrated the efficiency of single-step IJI to deliver the ink to the interlayer location through the porous cathode scaffold. Cobalt oxide has been shown to act as a sintering aid for ceria-based solid solutions by increasing the shrinkage rate at relatively low calcination temperatures [59]. The effect is generally assigned to the formation of a grain-boundary film, providing improved densification routes [60]. Nano-powders possessing a high specific surface area, and thus a high driving force for sintering, experience densification onset at lower temperatures, depending on the Co_x_O_y_ doping levels. According to Jud et al., the onset of an enhanced shrinkage rate is observed above 780 °C, with the maximum shrinkage rate at temperatures above 880 °C [61]. Prasad et al. reported the temperature of the maximum shrinkage rate at 820 °C for powders synthesised by the deposition–precipitation method [62], which is relevant to the method presented in this work. This can explain why a drop in the Ohmic resistance was observed only at 800 °C, where an effective densification of the interlayer provided additional ion conductive paths, reducing the overall Ohmic resistance.

The synergetic effect of doped ceria and Co_x_O_y_ co-infiltration led to enhanced densification of the GDC interlayer as well as catalytic contribution of the interface region to the ORR at 800 °C. Despite the non-percolating nature of GDC-Co_x_O_y_ nano-decoration, a clear reduction in the polarisation resistance was observed at 700 °C, as evidenced by EIS. A similar effect was reported by Samson et al. [56], who produced cathodes by infiltration of Co_x_O_y_ into the porous backbone of GDC and measured an *R_p_* of 0.27 Ω cm^2^ at 600 °C in air. We speculate that the cause of the observed electrochemical enhancement in this work was related to the nature of the ion exchange between the LSCF bulk cathode and the GDC-Co_x_O_y_ nano-decoration. A possible mechanism was proposed by Ding et al. [31], who suggested that the oxygen vacancies (V_o_^••^) prefer to occupy sites next to Co due to the strong Co–V_o_^••^ binding energy. Therefore, oxygen vacancies’ diffusion from LSCF to non-percolating GDC-Co_x_O_y_ nanoparticles could enhance the catalytic activity, as observed in the EIS data. The process would reduce the V_o_^••^ concentration in the LSCF sub-surface, hence lowering the surface charge and suppressing Sr out-diffusion and segregation. In conformation, an experimental study by Druce et al. [63] demonstrated a substantial enhancement of the surface exchange coefficient (k*) of GDC when coated with Co_3_O_4_. Treatment with Co nitrate was shown to produce an enhancement of k* by approximately an order of magnitude at 700 °C (k*_untreated GDC_ = 1.1 × 10^−8^; k*_coated GDC_ = 1.5 × 10^−7^).

Although studies on cathode nano-engineering by infiltration have been widely published in recent years, to the best of our knowledge, this is the first report on post-processing modification of the doped-ceria interlayer in commercial AS-SOFCs. The simplicity of the combined (doped ceria plus Co_x_O_y_) inkjet printing infiltration provides encouraging answers on how to scale up the infiltration procedures via a non-disruptive and cost-saving technique. 

## 5. Conclusions

LSCF cathodes and GDC interlayers of commercial anode-supported SOFCs were successfully infiltrated with doped ceria and Co_x_O_y_ inks via industrial inkjet printing. The infiltration was done in a single-step procedure without vacuum treatment or intermediate high-temperature calcinations. Relatively low infiltration loading (~5 wt%) led to substantially enhanced electrochemical performance. The infiltrated GDC interlayers were densified, which led to a reduction in the overall Ohmic resistance. An improvement in the polarisation resistance was also registered at 700 °C and assigned to the synergetic catalytic contribution of Co_3_O_4_/doped ceria infiltration causing beneficial surface modification. Inkjet printing infiltration was demonstrated to be a feasible, non-disruptive and cost-effective technology for infiltration nano-engineering of SOFCs. The combination of high printing speeds, accurate drop delivery, ink conservation and availability of industrial multi-nozzle systems presents an opportunity for scaling up IJI to a commercial-level SOFC technology.

## Figures and Tables

**Table 1:** Solution infiltration inks’ viscosities and jetting parameters.

Ink	Cation Concentration, M	Viscosity, cP	Opening Time, µs	Pressure, mbar	Drop Volume, nL	Drop Velocity, ms^−1^
YDC	0.75	4.8	240	200	57	1.5
Co_x_O_y_	0.75	4.3	230	225	62	1.6

## Data Availability

Not applicable.

## Supplementary Materials

The following are available online at https://www.mdpi.com/article/10.3390/nano11113095/s1, Figure S1: Drop visualization optimisation of YDC ink jetting - (a) drop velocity and (b) drop volumes vs. the print head working pressure and nozzles opening time.
