# Supplementary material for: Inkjet Printing Infiltration of the Doped Ceria Interlayer in Commercial Anode-Supported SOFCs
Source: Nanomaterials (Basel). 2021 Nov 16;11(11):3095. doi: 10.3390/nano11113095 (PMC8622447; doi:10.3390/nano11113095)
Supplement: Supplementary file 1 [file nanomaterials-11-03095-s001.zip › nanomaterials-1430217-supplementary.pdf]

Supplementary information for

"Inkjet printing infiltration of doped ceria interlayer in commercial anode-supported SOFCs"

R. I. Tomov, T. B. Mitchel-Williams, E. Venezia, M. Kawalec, M. Krauz[2], R. V. Kumar, B. A. Glowacki

Jetting optimization

The infiltration experiment was performed using 16 nozzle Domino Macrojet print head with 100 μm nozzle orifice. This commercial electromagnetic valve printing technology is reliable for use with high throughput volume of inks, robust and easy to maintain as well as compatible with wide range of ink chemistries. To ensure accurate and precise jetting, drop visualisation and optimisation of the inks was performed. The pressure applied to the fluid reservoir and the opening time of the nozzle orifice, were both varied in order to achieve repeatable jetting of well-defined drops. The system for visualisation consisted of a collinear LED strobe to backlit the drops in flight, and a camera (Stingray F-125B, Allied Vision Technologies) fitted with a zoom lens (ML-Z07545, Moritex). The nozzle and the camera shutter were precisely triggered with increasing delay times in order to image the entire jetting process. The images were then analysed using in-house developed software that quantified the drop volumes and velocities. This enabled optimisation of the jetting parameters in order to produce desired drop formation and to ensure equivalent quantities of ink were uniformly distributed on the surface. Drop visualization optimization enabled ink jetting parameters to be tailored in such a way that at certain jetting velocity each triggering event resulted in a single drop, without formation of satellite drops or splashing effects. Figure 1 illustrates how the working parameters of the print head influence the drop volumes and the drop velocities. It can be seen that higher pressures and longer opening times lead to higher drop volumes. The velocities were predominantly dependent on pressure while the opening time had a weaker effect.

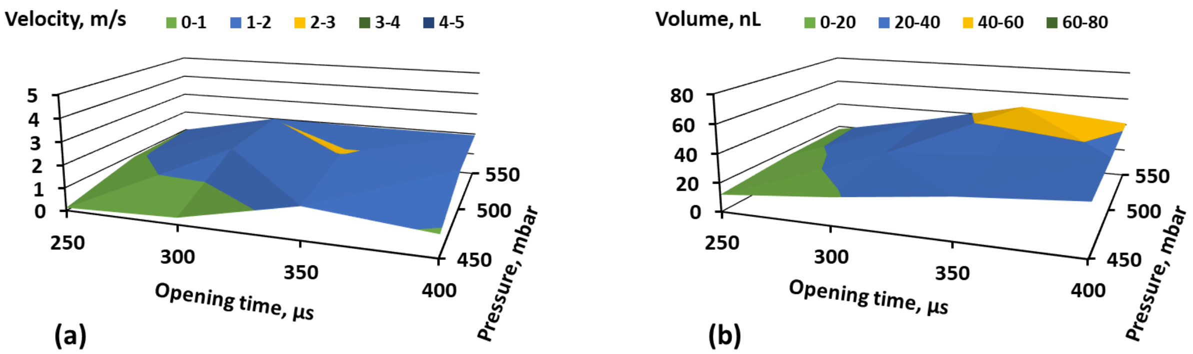

Figure S1. Drop visualization optimisation of YDC ink jetting - (a) drop velocity and (b) drop volumes vs. the print head working pressure and nozzles opening time.
